# Traditional Chinese medicine improves myasthenia gravis by regulating the symbiotic homeostasis of the intestinal microbiota and host

**DOI:** 10.3389/fmicb.2022.1082565

**Published:** 2023-01-06

**Authors:** Mingli Zhao, Li Liu, Fanzhao Liu, Lei Liu, Zhijuan Liu, Yanli Gao, Jianxi Cao

**Affiliations:** ^1^Department of Cardio-Thoracic Surgery, The First Affiliated Hospital of Henan University of Traditional Chinese Medicine, Zhengzhou, China; ^2^Department of Thoracic Surgery, Henan Province Hospital of Traditional Chinese Medicine, Zhengzhou, China

**Keywords:** myasthenia gravis, traditional Chinese medicine, intestinal microbiota, immunological function, SCFAs

## Abstract

Myasthenia gravis (MG) is an autoimmune disease caused by autoantibodies that is dependent on T-cell immunity and complement participation and mainly involves neuromuscular junctions. In this study, 30 patients with myasthenia gravis were selected and divided into pretreatment (Case group) and posttreatment (Treatment group) and 30 healthy volunteers (CON group) were included. Among them, the treatment group was treated with Modified Buzhong Yiqi Decoction (MBZYQD), and the levels of antibodies such as AChR, Musk and Titin in blood and intestinal microbiota were compared before treatment (Case group), after treatment (Treatment group) and in healthy volunteers (CON group). The results showed that after treatment with MBZYQD, the antibody levels of AChR, MuSK, and Titin and the inflammatory factor level of IL-6, IL-1β, and IL-22 in MG patients decreased significantly and nearly returned to a healthy level. In addition, after treatment with MBZYQD, the diversity, structure and function of intestinal microorganisms in MG patients also recovered to a healthy level. At the phylum level, the relative abundance of Proteobacteria in the Case group increased significantly, accompanied by a significant decrease in the relative abundance of Bacteroides compared with that in the CON group, the relative abundance of Proteobacteria and Bacteroides in the Treatment group was similar to that in the CON group. At the genus level, the relative abundance of *Shigella* in the Case group was significantly increased, accompanied by a significant decrease in the relative abundance of *Prevotella*, and the relative abundance of *Shigella* and *Prevotella* in Treatment group was similar to that in the CON group. Moreover, the fluorobenzoate degradation pathway (KO00364) was significantly increased in the Case group, while this pathway was significantly decreased in the Treatment group. In conclusion, MBZYQD can improve the immune function of the host by regulating the diversity, structure and function of the intestinal microbiota to treat myasthenia gravis.

## Introduction

Myasthenia gravis (MG) is an autoimmune mediated disease (Gilhus and Verschuuren, 2015). Its etiology includes environmental factors and genetic factors. At present, antibodies to acetylcholine receptor (AChR), muscle specific receptor tyrosine kinase (MuSK), and Rankine receptor (RyR) are known (Zhao et al., 2008; Sieb, 2014; Müllges and Stoll, 2019). These antibodies can interfere with the aggregation of AChR and affect the function of AChR and the signal transmission of nerve-muscle junctions, resulting in the failure of nerve-to-muscle action potential transmission (Gomez et al., 2010). Its main clinical manifestations are skeletal muscle weakness, fatigue, aggravation after activity, ptosis, diplopia, dysphagia, unclear articulation, and weak mastication (Zhao et al., 2011).

There are a large number of microorganisms in the human intestine, and they gradually form a complex relationship of mutualism with humans (Hugon et al., 2015). The intestinal microbiota play a variety of role; they are not only responsible for digesting and absorbing nutrients, but also play an important role in regulating the proliferation and differentiation of epithelial cells and resisting the invasion of pathogens (Soderholm and Pedicord, 2019; Rohr et al., 2020; Stacy et al., 2021). Moreover, the intestinal microbiota play an important role in promoting the occurrence and development of host innate immunity and its acquired immune system (Thaiss et al., 2016; Pascal et al., 2018). Studies have shown that there are obvious defects in the development and maturation of the spleen, mesenteric lymph nodes and intestinal-associated lymphoid tissues of sterile mice, and the administration of microbiota or some metabolites of microbiota can induce the above tissues to become normal, which indicates that intestinal microbiota are indispensable in the maturation of the host immune system (Kamada et al., 2013). Moreover, the immunosuppressive effect of intestinal microbiota on T lymphocytes is mainly reflected in the two cell helper T lymphocytes - helper T cells and regulatory T cells (Yang et al., 2020). Regulatory T cells in the intestine have the anti-inflammation functions, maintaining the immune tolerance of the body to its own harmful substances, and preventing the occurrence of autoimmune disorders in the host (Lan et al., 2007; Gao et al., 2016; Elmadfa and Meyer, 2019).

Previous research found that compared with a normal group, the diversity of microorganisms in the feces of patients in an MG group decreased as a whole, and the structure of the microbial community also changed, especially the SCFA level in the feces of patients in the MG group, which decreased significantly (Qiu et al., 2018; Liu et al., 2021). The same study also found that the Firmicutes/Bacteroides ratio (F/B ratio) in the intestinal microbiota of MG patients was significantly lower than that of the healthy control group, and the F/B ratio can be regarded as a pro-inflammatory environment. The inflammatory microbiota may cause damage to intestinal epithelial cells and trigger an immune response, eventually leading to the occurrence of various autoimmune diseases and an imbalance in the immune system (Qiu et al., 2018; Rinaldi et al., 2018). Other research explored the fecal microbiota of MG experimental mice (experimental autoimmune myasthenia gravis, EAMG) and healthy mice: At the phylum level, the ratio of Tenericutes/Verrucomicrobiota in the experimental autoimmune myasthenia gravis (EAMG) model group significantly decreased compared with that in the healthy control group, and Tenericutes/Verrucomicrobiota ratio partially recovered after probiotic treatment. At the family level, Lachnospiraceae decreased significantly in the EAMG group, and the ratio of Ruminococcaceae/Lachnospiraceae increased significantly. After treatment with probiotics, the ratio of Ruminobacteriaceae to Lachnospiraceae decreased significantly (Rinaldi et al., 2019). Additionally, Zheng et al. colonized germ-free mice with MG microbiota and healthy microbiota. The MG microbiota mice exhibited markedly impaired motor performance compared with healthy microbiota mice, and this insufficiency could be reversed by cocolonization of germ-free mice with MG microbiota and healthy microbiota mice. The above experimental results further support the correlation between gut microbiota and MG (Zheng et al., 2019).

In recent years, the role of intestinal microbiota in the immune system and their correlation with autoimmune diseases have been gradually explored. Intestinal microbiota may play a role in the occurrence and development of MG by affecting key factors in the anti-inflammatory process (Rinaldi et al., 2019). Traditional Chinese medicine (TCM) treatment is now considered a new immunomodulatory tool and a potential treatment that may be used to improve the symptoms of MG. Traditional Chinese medicine believes that MG belongs to the category of “flaccidity syndrome.” Most studies also show that the method of invigorating the spleen and replenishing qi is the key to the treatment of MG, and most of them use Buzhong Yiqi Decoction as the base prescription, which is treated according to syndrome differentiation, and has achieved remarkable effects (Jiang et al., 2018; Li et al., 2021; Zong et al., 2021). In our clinical work, we found that MBZYQD has a significant effect in the treatment of myasthenia gravis. Its main ingredients are nine medicines, including *Astragalus*, *Codonopsis*, *Atractylodes macrocephala*, *Cohosh*, and *Bupleurum chinense*, et al., among which the main active ingredients are flavonoids, including quercetin, luteolin, kaempferol, and naringenin. These active ingredients have anti-inflammatory and antioxidant effects (Imran et al., 2019; Kracht et al., 2020). However, the effect of MBZYQD on the intestinal microbiota of myasthenia gravis is unknown. By using the hypervariable marker sequence of the V3-V4 region of the 16S rRNA gene, this study established the systematic pattern spectrum of the intestinal microbial population, compared the differences in fecal microbiota between MG patients and a healthy control group, as well as MG patients treated with MBZYQD before and after treatment, and discussed the possible mechanism of traditional Chinese medicine in the treatment of MG to lay a solid foundation for the discovery of new diagnosis and treatment methods.

## Materials and methods

### Case source

MG patients who met the inclusion criteria in the cardiothoracic surgery clinic of the First Affiliated Hospital of Henan University of Traditional Chinese Medicine were selected, and a sample size of *n* = 90 was obtained according to the case–control study design. That is, 30 MG patients (before (Case group, *n* = 30) and after treatment (Treatment group, *n* = 30)) and 30 healthy volunteers (CON group, *n* = 30) were selected in this study (Volunteers with one of the following conditions were excluded: liver and /or kidney disease, mental disease, tumor, gastrointestinal disease, metabolic disease or any other disease that might affect the results of the study. Antibiotics, glucocorticoids, anti-obesity agents, monoclonal drugs, hypoglycemic agents or probiotics within 3 months were excluded).

### Ethical review

The clinical research plan met the ethical standards of and was approved by the clinical ethics committee of the scientific research project of The First Affiliated Hospital of Henan University of Traditional Chinese Medicine: HECABX-20210185.

### Diagnostic criteria

#### Inclusion criteria

According to the diagnostic criteria of myasthenia gravis in Western medicine, the condition of patients with myasthenia gravis is in a stable stage. In line with the diagnostic criteria of TCM syndrome differentiation, the disease belongs to deficiency of spleen and stomach qi. The age of the patients with myasthenia gravis in Henan was between 18 and 70 years old. No patients had thymoma. The test design was explained to the patients to help them understand the test contents, and the consent form was signed. Patients with myasthenia gravis who could cooperate with this treatment scheme and persist in treatment for 4 months were permitted to complete the detection of the main observation indices. Those who met the above conditions could be selected.

TCM syndrome was refeenced from the National Standard of the People’s Republic of China Terms for Clinical Diagnosis and Treatment of Traditional Chinese Medicine-Symptoms, issued by the State Administration of Technical Supervision and from the Guidelines for Diagnosis and Treatment of Common Diseases in Internal Medicine of Traditional Chinese Medicine - Western Medicine Diseases issued by the Chinese Society of Traditional Chinese Medicine in 2008. Spleen and stomach qi deficiency syndrome was characterized by main symptoms, including drooping eyelids, light twilight, limb weakness, and dysphagia, chewing difficulty or chewing weakness; secondary symptoms, including a lack of qi and laziness, stuffiness and shortness of breath in the chest, choking after drinking water, inability to lift the neck, loss of appetite, abdominal distension, fatigue, body fatigue, sallow complexion, and loose stool. Tongue pulse was characterized by the tongue being light and fat, with tooth marks on the edge, with thin and white fur, and a weak pulse. TCM syndrome differentiation required that the main symptoms be and at least one secondary symptom was present, along with the tongue pulse meeting the described criteria.

#### Exclusion criteria

Exclusion criteria included the following: MG symptoms did not conform to TCM syndrome types (spleen stomach qi deficiency syndrome); myasthenic crisis and cholinergic crisis had occurred in the last 6 months; for women, being pregnant or lactating; having experienced the surgical removal of thymoma; being diagnosed with a serious mental illness or being unable to cooperate with the clinical investigation; having complications of serious heart, kidney, blood, or other important organ diseases; being allergic to drugs that might be used in this study; and having recently participated in other clinical studies.

### Treatment plan

Thirty patients with myasthenia gravis of spleen stomach deficiency of vital energy type were treated with MBZYQD (drug composition: *Astragalus membranaceus* (reuse), *Atractylodes macrocephala*, *Radix Pseudostellariae*, *Poria cocos*, *Angelica sinensis*, *Tangerine peel*, *Bupleurum*, *Cohosh*, *Roasted licorice*, etc.), which was uniformly decocted by the decoction machine in the preparation room of the First Affiliated Hospital of Henan University of Traditional Chinese Medicine, 1 dose/day, 2 times/day).

### Research period and sample collection

**Figure 1:**
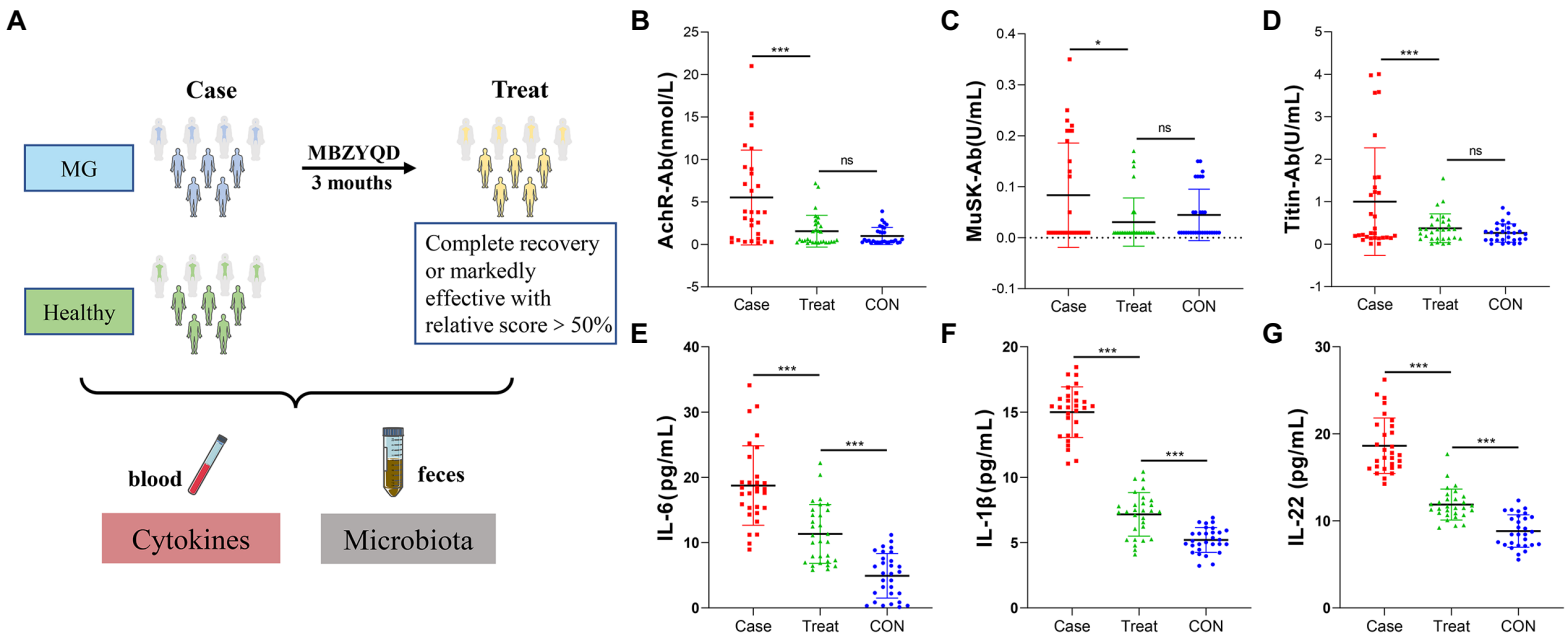
Experimental framework and Blood biochemical indexes. **(A)** Experimental framework. **(B)** AChR antibody content. **(C)** Musk antibody content. **(D)** Titin antibody content. **(E)** IL-6 content. **(F)** IL-1β content. **(G)** IL-22 content. Asterisks denote significance (*p < 0.05; **p < 0.01; ***p < 0.001).

The study period was 3 months (The overall framework of this study is shown in Figure 1A). The index collection time point at which feces and blood samples were collected occurred once the MG patients were enrolled (i.e., before the intervention of MBZYQD). Within 3 months of treatment, it was indicated that it was effective or cured according to the efficacy evaluation criteria. Combined with the absolute and relative score table of myasthenia gravis, feces and blood samples were taken again for patients with a relative score of more than 50%. The absolute and relative clinical scoring criteria for myasthenia gravis were determined in Supplementary material S1. Fecal samples were collected once the healthy control group was enrolled. Blood test items mainly included AChR antibody, MuSK antibody, Titin antibody levels and inflammatory factor levels (IL-6, IL-1β, and IL-22). The clinical symptoms of myasthenia gravis patients before and after treatment are shown in Table 1.

**Table 1:** The clinical symptoms of myasthenia gravis patients before and after treatment.

Variables	Case group	Treatment group	*p* value
Gender (Female)	15 (50%)	15 (50%)	NA
Age-years	59.47 ± 13.33	59.47 ± 13.33	NA
ASMG	13.56 ± 9.36	3.52 ± 3.06	0.001
RSMG (Total efficiency)	NA	93.86 ± 5.62	NA
MGFA	9.56 ± 4.23	2.36 ± 1.56	0.025
ADLMG	6.52 ± 3.17	1.65 ± 1.03	0.032

Note: ASMG, Absolute score of myasthenia gravis; RSMG, Relative score of myasthenia gravis; MGFA, Myasthenia gravis foundation of America; ADLMG, Activities of daily living of myasthenia gravis.

### Extraction and database construction of fecal genomic DNA

From the CON, Case and Treatment groups of fecal samples, 10, 17, and 13 samples were randomly selected for 16S rRNA gene sequencing. Total genomic DNA samples of bacteria in feces were extracted using the fecal DNA Extraction Kit (MP biomedical, Santa Ana, CA, United States) according to the manufacturer’s instructions and stored at −20°C before further analysis. The concentration and quality of extracted DNA were measured by the NanoDrop ND-1000 spectrophotometer and agarose gel electrophoresis, respectively. The V3-V4 region of the bacterial 16S rRNA gene was amplified by PCR using forward primer (5′-ACTCCTACGGGGAGGGCAGCA-3′) and reverse primer (5′-GGACTACHVGGTWTCTAAT-3′). Illumina’s TruSeq Nano DNA LT Library Prep Kit was used to prepare the sequencing library.

### Bioinformatics analysis

#### Alpha and beta diversity analysis

In this study, the alpha diversity index and beta diversity index were used to characterize the diversity of species within and between habitats respectively, to comprehensively evaluate their overall diversity. Alpha diversity was calculated as follows: Using QIIME2 (2019.4) and the ggplot2 package in R language, calculate the alpha diversity index (mainly including Chao1, observed species, Shannon, Simpson, Faith’s, PD Pielou’s evenness and Good’s coverage) was calculated according to the ASV/OTU table that was not flattened to detect biological diversity. Then, the R language was used to draw the specaccum species accumulation curve for the total number of ASVs/OTUs corresponding to each sample in the ASV/OTU abundance matrix to test whether the sample size of this study was sufficient. Beta diversity was calculated as follows: The vegan package in the R script was used for NMDS analysis. Through the dimensionality reduction decomposition of the sample distance matrix, the data structure was simplified, and the distribution characteristics of samples were described at a specific distance scale to show the composition differences of microbial communities.

#### Species composition analysis

Called the “QIIME taxa barplot” command in QIIME2, statistical calculations were performed on the feature table after removing singletons; the composition distribution of each sample at the phylum and genus levels was visualized; and the analysis results were presented in a histogram. In addition to showing the formation of taxonomic composition in the form of a tree diagram, in this study, ggtree was used as an evolutionary tree to show the position of ASV/OTU in the evolutionary tree and the evolutionary distance between them and to reflect their composition, abundance, taxonomy and other information through a heatmap and histogram (Wang et al., 2020).

#### Species difference and standard species analysis

After exploring the differences in microbial community composition, we also need to know which species are mainly caused by these differences. LEfSe analysis is a difference analysis method that which can directly analyze the differences of all classification levels at the same time. At the same time, LEfSe puts more emphasis on finding robust differential species between groups, namely marker species (biomarkers). One of its characteristics is that it is not only limited to analyzing the differences in community composition in different sample groups but can also go deep into different subgroups and identify the marker microbial groups that are consistent in different subgroups. At present, it has been widely used in the fields of microbial amplification analysis and is especially suitable for finding biomarkers in medical research. To further compare the species composition differences between samples, the pheatmap package in R language was used to calculate the clustering results of each sample and each taxon, which is presented in the form of an interactive graph to display the distribution trend of species abundance of each sample. By default, the abundance data of the top 20 genera of average abundance were used to draw the heatmap.

#### Functional potential prediction

The above analysis focuses on the diversity and species composition of the microbiota. With the development of data analysis technology, we can refer to known microbial genome data to predict the composition of microbiota genes or functional units for samples with only microbiota marker gene sequencing data. The obtained functional units were used to obtain the abundance value of metabolic pathways according to the metabolic pathway database and R language analysis. Then, using the normalized functional unit abundance table, the R script was used to calculate the distance matrix in R and conduct PCoA. The PCoA coordinates of the sample points were output and drawn into a two-dimensional scatter diagram. Finally, after obtaining the abundance data of metabolic pathways, we attempted to identify the metabolic pathways with significant differences between groups.

### Statistical analysis

A completely randomized test design was used in the study. The significance of the difference between the means of the groups was determined by one-way ANOVA. Differences with *p* < 0.05 (*), *p* < 0.01 (**), and *p* < 0.01 (***) were considered to be statistically significant. The statistical calculations used in this study were performed with IBM SPSS 25.0.

## Results

### Blood biochemical indices

The results showed that the levels of AChR, MuSK, Titin and inflammatory factors (IL-6, IL-1β, and IL-22) in the Treatment group were significantly lower than those in the Case group, and there were no significant differences in the antibody levels of AChR, MuSK and Titin between the Treatment group and the CON group of healthy volunteers (Figures 1B–G).

### Species composition

**Figure 2:**
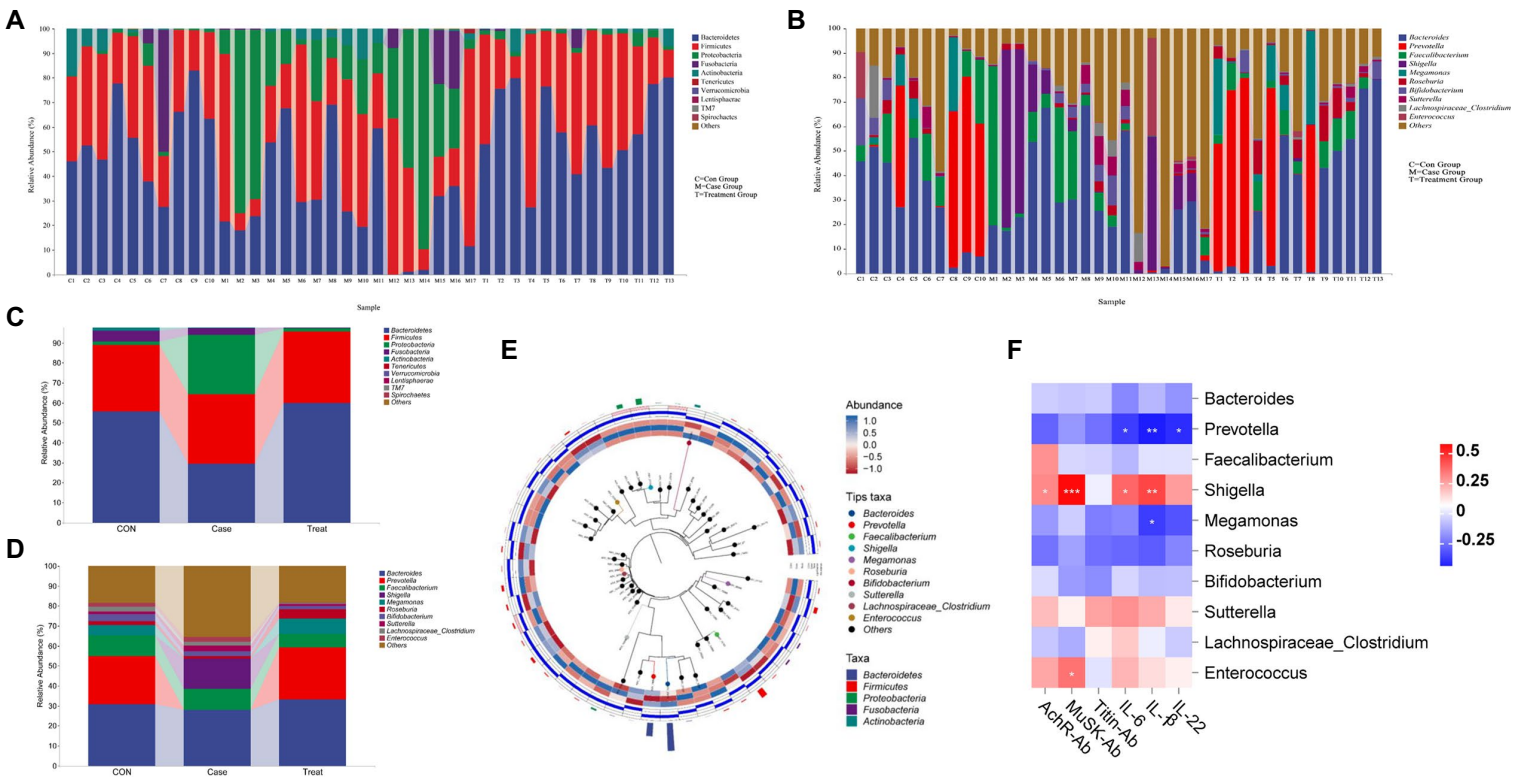
Species composition and association analysis. **(A,C)** Composition and distribution of intestinal microbiota at phylum level in Case group, Treatment group and CON group. **(B,D)** Composition and distribution of intestinal microbiota at genus level in Case group, Treatment group and CON group. **(E)** Phylotree evolutionary tree. **(F)** Correlation analysis of intestinal microbiota (Genus Level) and Cytokines. Asterisks denote significance (*p < 0.05; **p < 0.01; ***p < 0.001).

To further compare the species composition differences between samples and display the distribution trend of species abundance of each sample, a heatmap can be used for species composition analysis. At the phylum level, the relative abundance of Bacteroidetes in the Case group was significantly lower than that in the CON group, and the relative abundance of Proteus was significantly higher than that in the CON group. After treatment, the relative abundance of Bacteroidetes and Proteobacteria returned to the level of the CON group (Figures 2A,C). At the genus level, *Prevotella* and *Megamonas* were hardly detected in the Case group, but the relative abundance of *Shigella* was significantly higher than that in the CON group, and all bacteria returned to the level of the CON group after treatment (Figures 2B,D). Phylotree results show that *Bacteroides* and *Prevotella* are closely related, and both belong to Bacteroidea. They had high relative abundance in the Case group but low relative abundance in Treatment group and CON group. In addition, *Roseburia* and *Bifidobacterium* were closely related, and *Bifidobacterium* had a high relative abundance in the Treatment group (Figure 2E). The correlation analysis results of intestinal microbiota and cytokines showed that antibodies (AChR, MuSK, and Titin) and inflammatory factors (IL-6, IL-1β, and IL-22) were mainly positively correlated with *Shigella* and *Enterococcus*, but negatively correlated with *Prevotella*, *Bacteroides*, and *Bifidobacterium* (Figure 2F).

### Alpha and beta diversity

**Figure 3:**
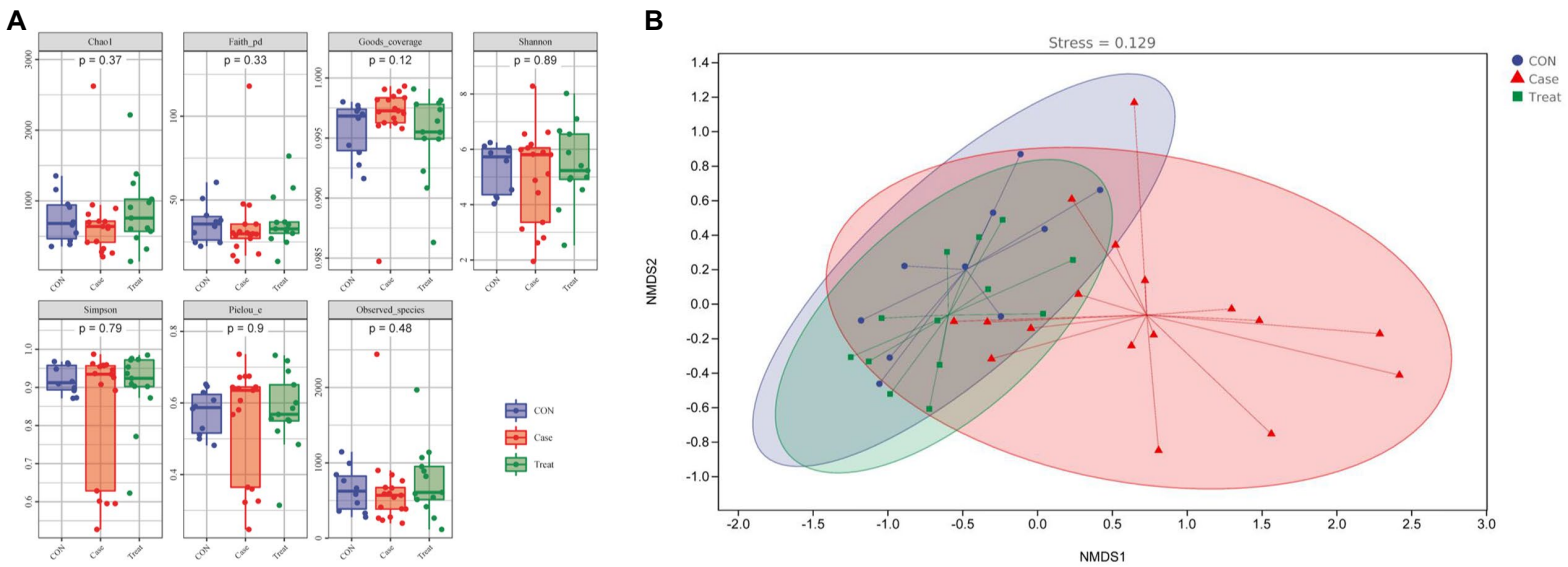
Alpha and beta diversity. **(A)** Alpha diversity indexes. **(B)** Nonmetric Multidimensional scaling (NMDS).

Alpha diversity refers to the indicators of richness, diversity and evenness of species in locally uniform habitats. It is also known as within-habitat diversity. In this study, the alpha diversity results showed that the Chao1 index, Faith PD index, Shannon index, Simpson and observed species index of the case group were lower than those of the CON group, while the coverage index was higher than that of the CON group. In the Treatment group, these alpha diversity indices were restored (Figure 3A). NMDS results showed that the dispersion degree among samples in the Case group was large. After MBZYQD treatment, the dispersion distance among samples in the Treatment group decreased, and there was a great difference between the Case group and the Treatment group, while the distribution degree of samples in the Treatment group was closer to that of the CON group (Figure 3B).

### Species differences and marker species

**Figure 4:**
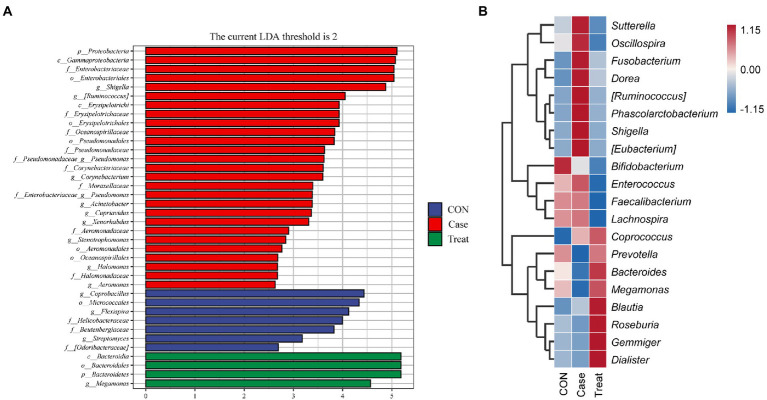
Species differences and marker species. **(A)** Lefse analysis. **(B)** Species composition heatmap at phylum level in Case group, Treatment group and CON group.

LEfSe analysis showed that *Proteobacteria*, *Coprobacillus*, *Bacteroidia*, *Bacteroidales*, and *Bacteroidetes* were the most significantly different bacteria in Case group, CON group and Treatment group, respectively (Figure 4A). To further compare the species composition differences between samples and display the distribution trend of species abundance of each sample, the species composition of the top 20 relative abundances was analyzed by a heatmap. The results showed that the microbiota of the Case group was significantly different from that of the CON group and Treatment group, such as *Sutterella*, *Oscillospira*, *Fusobacterium*, *Dorea*, *[Ruminococcus]*, *Phascolarctobacterium*, *Shigella*, and *[Eubacterium]*, while the relative abundance of *Prevotella*, *Bacteroides*, and *Megamonas* were significantly lower than those of the CON group. After treatment, the relative abundances of these bacteria returned to the normal level, reaching the level of the CON group (Figure 4B).

### KEGG metabolic pathway

**Figure 5:**
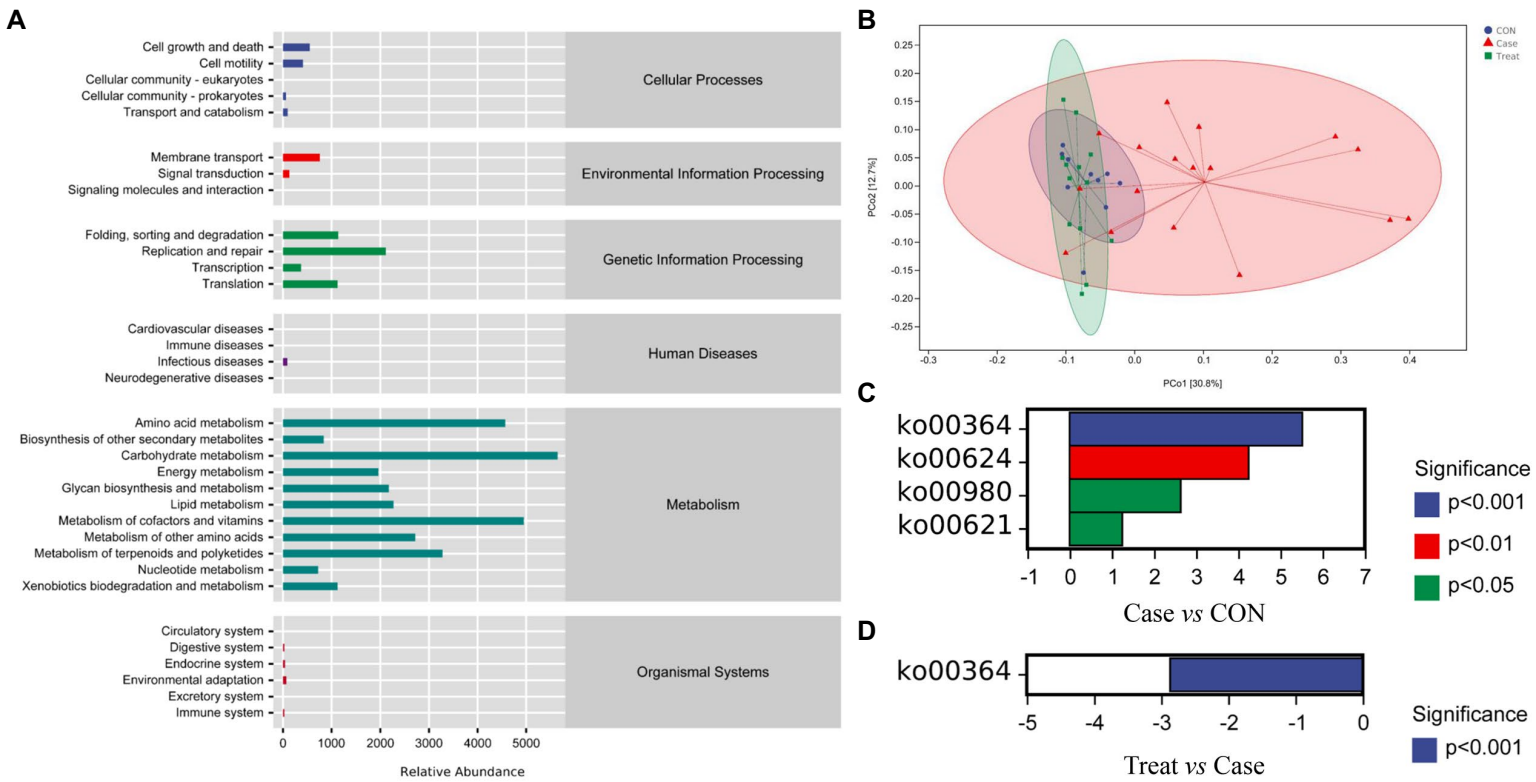
KEGG metabolic pathway. **(A)** KEGG metabolic pathway. **(B)** Functional unit PCoA analysis. Not: Each point in the functional unit PCoA analysis result diagram represents a sample, and points of different colors indicate different samples (groups). The percentage in the brackets of the coordinate axis represents the proportion of the sample difference data (distance matrix) that can be explained by the corresponding coordinate axis. The closer the projection distance of the two points on the coordinate axis, the more similar the functional composition of the two samples in the corresponding dimensions. **(C)** Metabolic pathway difference analysis between Case group and CON group. **(D)** Metabolic pathway difference analysis between Treatment group and Case group.

In this study, the microbiota was mainly involved in metabolic pathways, including amino acid metabolism, carbohydrate metabolism, coenzyme factor and vitamin metabolism (Figure 5A). The results of functional unit PCoA analysis were consistent with the NMDS results in the beta diversity analysis results; that is, the dispersion degree between samples in the Case group was large. After MBZYQD treatment, the dispersion ranges between samples in the Treatment group decreased, and there were great differences between the Case group and the Treatment group, while the distribution degree of samples in the Treatment group was closer to that in the CON group (Figure 5B). Further analysis of the differences in KEGG metabolic pathways among the three groups showed that the Case group significantly upregulated KO00364, KO00624, KO00980, and KO00621 compared with the CON group. The KO00364 pathway was significantly downregulated in the Treatment group compared with the Case group (Figures 5C,D).

## Discussion

Myasthenia gravis (MG) refers to an autoimmune disease in which the lesion occurs at the junction of the nerve and muscle, and the acquired neuromuscular junction transmission disorder is mediated by autoantibodies (Narayanaswami et al., 2021). The etiology of myasthenia gravis blepharoptosis is related to many factors (Mantegazza et al., 2018). At present, the commonly used treatment methods include cholinesterase inhibitors, immunosuppressants, thymectomy and so on (Farmakidis et al., 2018). However, because the disease is a chronic disease and has a long course, long-term use of the above drugs will produce a variety of adverse reactions, and surgical treatment is painful (Gajdos et al., 2012). Modern studies have found that there is a new cholinesterase inhibitor in MBZYQD (Liu et al., 2010; Cui et al., 2015). MBZYQD may affect the key factors in the anti-inflammatory process by regulating intestinal microbiota (Wang et al., 2022) and may play a role in the occurrence and development of MG (Li et al., 2021; Zong et al., 2021). However, it is unclear whether MBZYQD can improve myasthenia gravis by regulating intestinal microbiota. Therefore, this study analyzed the difference in intestinal microbiota in myasthenia gravis patients before and after MBZYQD treatment by high-throughput sequencing technology.

The results of this study showed that compared with the CON group, the diversity of microorganisms in the feces of patients in the MG group decreased as a whole, and the structure of the microbial community also changed. The study also found that the Firmicutes/Bacteroides ratio (F/B ratio) in the intestinal microbiota of MG patients was significantly lower than that of the healthy control group, and the F/B ratio can be regarded as a pro-inflammatory environment (Liu et al., 2021). The intestinal microbiota may cause damage to intestinal epithelial cells and trigger an immune response, eventually leading to the occurrence of various autoimmune diseases and an imbalance in the immune system (De Luca and Shoenfeld, 2019; Nakamoto et al., 2019; Di Tommaso et al., 2021). The view that the change in the F/B ratio is related to a series of autoimmune mediated diseases has also been supported by the research of Bibbò et al. (2017) and Shen et al. (2022). After MBZYQD treatment, the diversity and structure of the intestinal microbiota of MG patients recovered, and the ratio of Firmicutes/Bacteroides (F/B ratio) in the intestinal microbiota of MG patients also returned to normal.

Further analysis showed that the relative abundance of *Shigella* was significantly increased in Case group, the relative abundance of *Shigella* was restored in Treatment group, and the relative abundance of *Shigella* was closely related to that found in Alzheimer’s disease (AD), colitis and pancreatitis (Cattaneo et al., 2017; Li et al., 2020; Fan et al., 2021; Pivari et al., 2022). In addition, the relative abundance of *Prevotella* and *Bacteroides* was significantly reduced in the Case group and the relative abundance of *Prevotella* and *Bacteroides* was restored in the Treatment group, while *Prevotella* can use carbohydrates in the intestine to produce SCFAs, and the quantities and relative abundance of SCFAs can be considered biomarkers of health (Wu et al., 2011; Martínez et al., 2015; Yao et al., 2022). Atarashi et al. and Furusawai et al. found that SCFAs can directly regulate T cells to differentiate into CD4 + FoxP3+ T cells, change the phenotype of dendritic cells, induce the expression of Raldh1 in dendritic cells, promote the production of retinoic acid (RA), and induce the differentiation of CD4 + FoxP3+ T cells (Atarashi et al., 2013; Furusawa et al., 2015). Based on previous studies and the comparison between MG patients and healthy controls, it is speculated that the change in intestinal microbiota composition may lead to the lack or dysfunction of CD4 + FoxP3+ T cells induced by intestinal bacteria, which has a profound impact on immunity (Kwon et al., 2010; Chae et al., 2012). When the number of *Prevotella* and *Bacteroides* colonizing in the host decreases, the content of SCFAs in the intestine will be affected, and the number of CD4 + FoxP3+ T cells in the corresponding mucosal lamina propria will also be reduced, leading to an imbalance in the immune response (Mangalam et al., 2017; Shahi et al., 2019, 2020).

These results indicate that the increase in *Shigella* abundance and the decrease in *Prevotella* and *Bacteroides* abundance may be important reasons for the occurrence and development of myasthenia gravis. Moreover, the researchers fed EAMG rats mixed probiotic preparations of two lactobacilli and two *Bifidobacteria*. It was found that the mixed probiotic preparations could significantly reduce the clinical symptoms of EAMG rats by influencing the level of regulatory dendritic cells and inducing CD4+ T cells to transform into CD4 + Foxp3+ T cells. In addition, such preparations can reduce the level of AChR antibody and IFN-γ, TNF-α, IL-6, IL-17, and other immune factors levels, which is similar to the results of this study, indicating that intestinal microbiota can participate in the pathogenesis and development of MG (Chae et al., 2012). It can be inferred from the pathogenesis of myasthenia gravis and the immune regulation mechanism of intestinal microbiota that the immune response to stimulate T lymphocytes may be the key link of intestinal microbiota leading to MG (Chen and Tang, 2021).

Furthermore, in the analysis of KEGG metabolic pathways, it was found that the Case group significantly upregulated the pathways of fluorobenzoate degradation (KO00364), polycyclic aromatic hydrocarbon degradation (KO00624), metabolism of xenobiotics by cytochrome P450 (KO00980) and dioxin degradation (KO00621) compared with the CON group. The KO00364 pathway significantly downregulated in the Treatment group compared with the Case group. This result suggests that KO00364 may be the key metabolic pathway involved in myasthenia gravis. Previous reports have shown that this pathway is related to the severity of intestinal inflammation, such as Crohn’s disease (Montassier et al., 2015). In addition, the KO pathway related to the fluorobenzoic acid degradation pathway is often observed in the intestinal microbiota of patients with *Clostridium difficile* infection, and Proteobacteria bacteria involved in this pathway are significantly enriched (mainly including *Klebsiella*, *Escherichia coli*, *unclassified Enterobacteriaceae*, and *Salmonella*; Fujimoto et al., 2021). Subsequently, Fujimoto et al. significantly reduced the relative abundance of the KO pathway related to the fluorobenzoic acid degradation pathway and Proteobacteria (mainly including *Klebsiella*, *Escherichia coli*, *unclassified Enterobacteriaceae*, and *Salmonella*) in the intestinal microbiota of patients infected with *Clostridium difficile* through fecal microbiota transplantation (FMT; Fujimoto et al., 2021). This is consistent with the results of this study, indicating that MBZYQD can improve the intestinal environment, regulate the host immune level, and achieve intestinal microbiota host symbiosis and stability by reducing the relative abundance of Proteobacteria and the degradation pathway of fluorobenzoic acid in the intestine.

## Conclusion

Intestinal microbiota plays an essential role in the pathogenesis of myasthenia gravis. The results of this study show that the imbalance of the microbial community may be one of the causes of myasthenia gravis. Treatment with MBZYQD can adjust the diversity and structure of the intestinal microbiota and the function of the microbial community in MG patients. Reconstruction of the microbiota can further improve the immune function of the host, indicating that MBZYQD may play a role in the treatment of myasthenia gravis by regulating the intestinal microbiota and achieving intestinal microbiota host symbiosis and stability.

## Data availability statement

The datasets presented in this study can be found in online repositories. The names of the repository/repositories and accession number(s) can be found at: https://www.ncbi.nlm.nih.gov/genbank/, PRJNA895190.

## Ethics statement

The studies involving human participants were reviewed and approved by the clinical ethics committee of the scientific research project of The First Affiliated Hospital of Henan University of Traditional Chinese Medicine. Written informed consent to participate in this study was provided by the participants’ legal guardian/next of kin.

## Author contributions

MZ and LiL: data curation and writing — original draft. FL, LeL, and ZL: methodology. YG: visualization. JC: writing — reviewing and editing. JC: supervision, resources, and funding acquisition. All authors contributed to the article and approved the submitted version.

## Funding

This work was supported by the Special project for scientific research of traditional Chinese medicine in Henan Province (2019ZY1003). The funders had no role in the study design, data collection and interpretation or the decision to submit the work for publication.

## Conflict of interest

The authors declare that the research was conducted in the absence of any commercial or financial relationships that could be construed as a potential conflict of interest.

## Publisher’s note

All claims expressed in this article are solely those of the authors and do not necessarily represent those of their affiliated organizations, or those of the publisher, the editors and the reviewers. Any product that may be evaluated in this article, or claim that may be made by its manufacturer, is not guaranteed or endorsed by the publisher.

## Supplementary material

The Supplementary material for this article can be found online at: https://www.frontiersin.org/articles/10.3389/fmicb.2022.1082565/full#supplementary-material

## Abbreviations

MG: Myasthenia gravis
MBZYQD: Modified Buzhong Yiqi Decoction
SCFAs: Short chain fatty acids
AChR: Acetylcholine receptor
MuSK: Muscle specific receptor tyrosine kinase
RyR: Rankine receptor
F/B: Firmicutes/Bacteroides
TCM: Traditional Chinese Medicine
KEGG: Kyoto Encyclopedia of Genes and Genomes
NMDS: Non-metric multidimensional scaling
PCoA: Principal Co-ordinates analysis
FMT: Fecal microbiota transplantation
