# Supplementary material for: Traditional Chinese medicine improves myasthenia gravis by regulating the symbiotic homeostasis of the intestinal microbiota and host
Source: Front Microbiol. 2023 Jan 6;13:1082565. doi: 10.3389/fmicb.2022.1082565 (PMC9852828; doi:10.3389/fmicb.2022.1082565)
Supplement: Supplementary file 6 [file Data_Sheet_2.DOCX]

1. The clinical absolute and relative scoring standards (ASMG and RSMG) of myasthenia gravis were used to evaluate the efficacy:

The highest score of clinical absolute scoring method is 60 points. Each item is divided into 5 levels according to the severity of muscle weakness of each muscle group. 0 point is normal muscle strength (indicating that muscle strength is 5 levels). The higher the score is, the heavier the symptom of muscle weakness is [1]. The scoring method was evaluated from 8 aspects: upper eyelid weakness, upper eyelid fatigue, eye movement, upper limb fatigue, lower limb fatigue, facial muscle weakness, mastication and swallowing function, and respiratory muscle function. Among them, upper eyelid weakness, upper eyelid fatigue, eye movement, upper limb fatigue, and lower limb fatigue were evaluated according to the severity of 0-4 points, and bilateral scores were scored respectively, with a total score of 0-8 points for each item. Facial muscle weakness: 0-4 points. The function of swallowing and respiratory muscles can be divided into 5 grades from 2 angles. The degree of severity is 0~8 points, and 8 points are the most serious. Gastric tube and respiratory muscle should be retained to assist ventilation.

Relative clinical score = (absolute clinical score before treatment-absolute clinical score after treatment)/absolute clinical score before treatment * 100%. The criteria of clinical relative score were: 95%-100% recovery; 80%-95% were basically cured; 50%-80% effective; 25%-50% was improved< 25% is invalid.

2. Myasthenia gravis severity scale (MGFA)

According to the muscle weakness of each muscle group, there are 13 directions, including eyelid, diplopia, eye movement, facial muscle, swallowing, phonation, grip strength, upper limb and lower limb muscle strength. Each direction is divided into 4 grades according to the severity. 0 is normal, 1 is mild, 2 is moderate, and 3 is severe. The total score is 39. The higher the score, the more severe the symptom is.

3. Activities of daily living of myasthenia gravis (ADLMG)

To assess the level of daily living of patients with myasthenia gravis. Evaluated from the aspects of speaking, chewing, swallowing, breathing and other aspects of life, 0~3 points for each angle, 0 point is normal, and 3 points indicates that the symptoms are severe and need external help to complete. A total of 24 points, 24 points indicate that life completely needs help from others, requires gastric tube feeding, and respiratory muscles assist ventilation.

[1] Xu et al. Neuroimmunology [M]. Hubei Science and Technology Press, 2000:139
