# Supplementary figures and images for: Traditional Chinese medicine improves myasthenia gravis by regulating the symbiotic homeostasis of the intestinal microbiota and host
Source: Front Microbiol. 2023 Jan 6;13:1082565. doi: 10.3389/fmicb.2022.1082565 (PMC9852828; doi:10.3389/fmicb.2022.1082565)

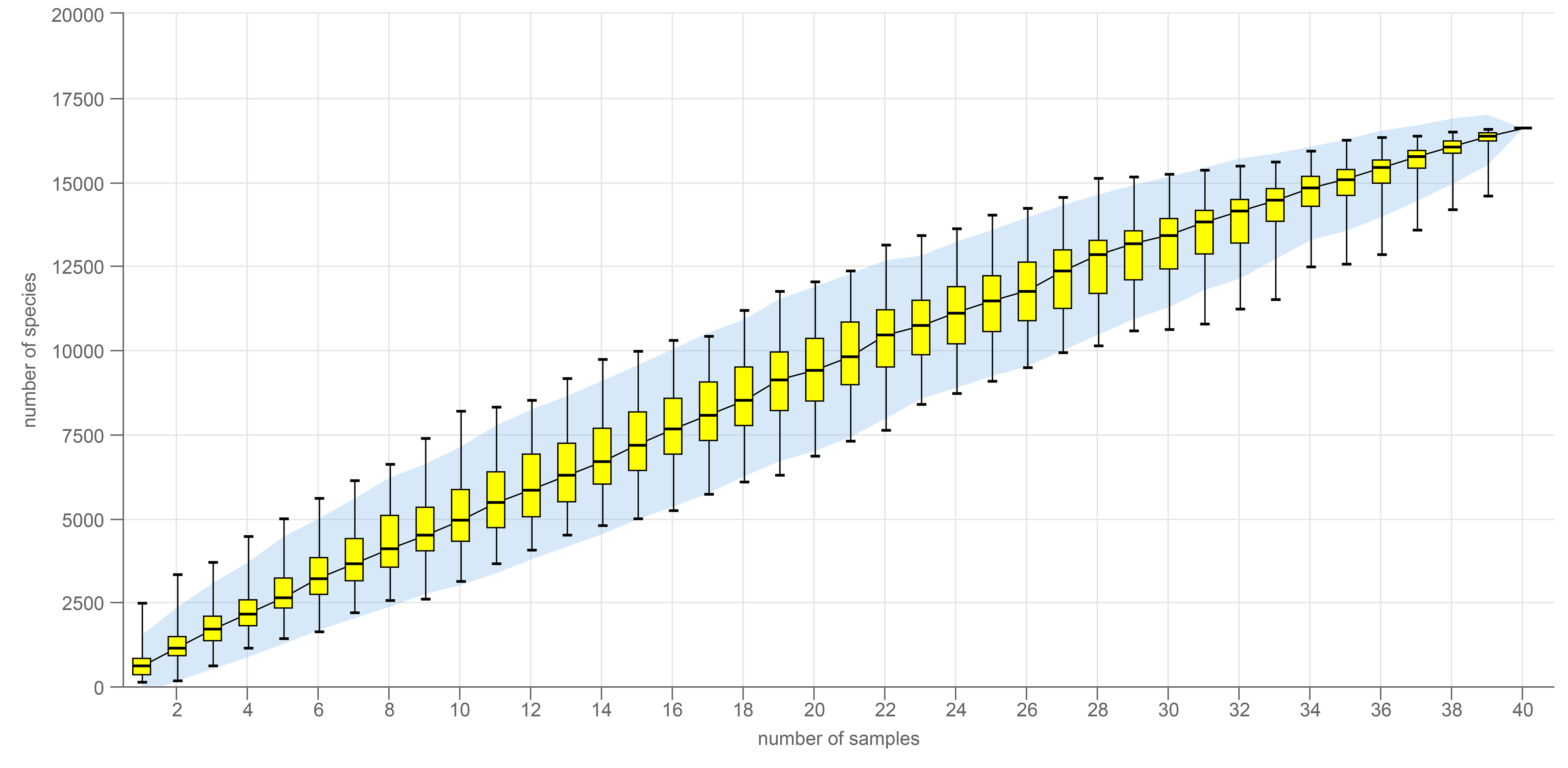

Supplement: Supplementary file 7 [file Image_1.TIF]
